# The Acceptability and Initial Effectiveness of “Space From Money Worries”: An Online Cognitive Behavioral Therapy Intervention to Tackle the Link Between Financial Difficulties and Poor Mental Health

**DOI:** 10.3389/fpubh.2022.739381

**Published:** 2022-04-14

**Authors:** Thomas Richardson, Angel Enrique, Caroline Earley, Adedeji Adegoke, Douglas Hiscock, Derek Richards

**Affiliations:** ^1^Richardson Psychological Consultation Limited, The Psychotherapy Practice, Southampton, United Kingdom; ^2^Clinical Research & Innovation, SilverCloud Health, Dublin, Ireland; ^3^E-Mental Health Research Group, School of Psychology, University of Dublin, Trinity College Dublin, Dublin, Ireland

**Keywords:** debt, financial difficulties, poverty, depression, anxiety, mental health, computer-based CBT, online CBT

## Abstract

**Background:**

Previous research has shown a strong relationship between financial difficulties and mental health problems. Psychological factors such as hope and worry about finances appear to be an important factor in this relationship.

**Objective:**

To develop an online based psychological intervention (Space from Money Worries) to tackle the psychological mechanisms underlying the relationship between poor mental health and financial difficulties, and to conduct an initial evaluation of the acceptability and preliminary efficacy of the intervention.

**Materials and Methods:**

30 participants accessing Increasing Access to Psychological Therapies (IAPT) services completed GAD-7 to measure anxiety and PHQ-9 to measure depression upon signing up to the online intervention and again 4 to 8 weeks after this. Participants also completed a measure of perceived financial distress/wellbeing and a “Money and Mental Health Scale” constructed for the evaluation.

**Results:**

Overall, 77% (*n* = 23) completed the intervention and follow-up assessments. Intent to Treat Analysis showed that there were statistically significant improvements in symptoms of depression, anxiety, improved perceived financial wellbeing and reduced scores on the money and mental health scale. The vast majority of participants rated each module positively.

**Conclusions:**

Space from Money Worries appears to be acceptable and may lead to improvements in mental health, perceived financial wellbeing and a reduced relationship between financial difficulties and poor mental health. However, future research with a larger sample and a control group are needed to confirm that these changes are due to the intervention.

## Introduction

A large body of research has shown a relationship between financial difficulties and mental health problems. Lower socio-economic status is linked to a greater risk of psychiatric disorders in prospective cohort studies (1), and socioeconomic deprivation is linked to higher suicide rates (2). Poorer living standards increase the risk of depression over time (3). Research with a range of different populations shows that those in debt are more than three times as likely to experience mental health problems, and are at a greater risk of specific difficulties including depression, substance use problems and suicide attempt and completion (4). A recent study showed a 2.5 fold risk of completion of suicide across the course of a year for those in debt (5).

In a sample of university students, greater financial difficulties such as struggling to pay electricity bills and having to borrow money is linked with more symptoms of anxiety, psychosis, eating disorder and alcohol problems over time (6–8). This research also showed that that this relationship can work both ways with mental health problems leading to worsening finances as well as financial difficulties leading to poorer mental health over time (6, 7). A recent longitudinal study also showed that debts increased the risk of developing a common mental health problem over the course of a year, but those with existing mental health problems were also more at risk of being unable to pay back debts over the course of the year (9). Qualitative research with those with Bipolar Disorder has similarly identified a theme of a “Vicious Cycle” between money and mental health problems (10).

Research shows that the risk and severity of common mental health disorders such as depression is increased by other specific financial difficulties such as low income (11), being on benefits (12, 13), job insecurity and unemployment (14, 15), bankruptcy (16), levels of financial strain (17), having to borrow money (11), use of short-term “payday loans” (18), having a utility disconnected (11) and having to go without items such as clothes, heating, or socializing (13).

There is potential overlap between those accessing mental health and debt advice services: One study screened those in contact with a debt advice charity and found that 65% scored high enough on measures of depression and anxiety to suggest that a referral for mental health support was warranted (19). The impact of financial difficulties on mental health has led to suggestions that assistance with financial difficulties within mental health services may improve recovery rates (20).

The financial impact of the COVID-19 pandemic is likely to have a considerable impact on mental health at a population level, given that a systematic review of 100 papers concluded that recessions increase the prevalence of common mental disorders, substance use problems, and suicidal behavior (21). This appears to be due to the impact of economic factors such as less income, unmanageable debts and unemployment (21). Research during the COVID pandemic has showed that reduced income or work due to COVID-19 increases symptoms of depression and anxiety (22), and COVID-related financial difficulties are linked to an increase risk of suicidal thoughts or thoughts of self-harm (23).

Research has shown the role of psychological factors in the role between financial difficulties and poor mental health. The impact of debt on anxiety appears to be moderated by stress about debt (24). Greater subjective concern and stress about finances is linked to poorer mental health (7, 25), and worries about debt predicts greater symptoms of depression over time, better than other financial variables such as income and employment status (26). One study showed differences in subjective financial wellbeing between individuals and found that this impacted overall wellbeing (27). Another found that questions about subjective financial wellbeing, such as worry about finances, were more strongly related to mental health than objective measures of financial hardship, such as being unable to pay the bills, in the general population (28), a finding which has been replicated in those with a diagnosis of Bipolar Disorder (13). Furthermore, hope is found to partially mediate the impact of subjective financial hardship on stress, depression and wellbeing, whilst shame partially mediates the impact on anxiety (28). Hopelessness also partially mediates the impact of debt on suicidal ideation (29). A systematic review concluded that self-esteem, active coping and sense of agency have a role in linking financial hardship with poor mental health (30). It has been suggested by a UK based think-tank, which focuses on money and mental health, that psychological approaches can address the causes and consequences of financial difficulties, and that work on changing thinking patterns and coping strategies around finances may “*help to provide a lasting fix to the vicious cycle of financial difficulty and mental health problems*” [(31), p.7]

Research has evaluated the impact of Cognitive Behavioral Therapy (CBT) in those who are unemployed, with mixed results about effectiveness (32). There has also been some research on community based interventions such as debt advice, social prescribing and labor market programmes, however there is not much evidence about the impact of this on mental health (33). Internet-delivered CBT (iCBT) has been shown by several meta-analyses to be effective for symptoms of depression and anxiety (34–38), and represents a way to increase access to psychological interventions. Trials also show the cost-effectiveness of online interventions over up to 12 months (39).

To the authors knowledge, only one previous online intervention has used a psychological approach to mitigate the impact of financial difficulties on mental health, specifically focusing on the stress associated with debt (40).

This study therefore describes the development of an online psychological intervention to try to address the link between financial difficulties and poor mental health. It attempts to assess the suitability of a digital CBT intervention for financial difficulties and mental health in the context of the Improving Access to Psychological Therapies (IAPT) program, which is a stepped care approach for the treatment of common mental health disorders in England provided by the National Health Service (NHS). The primary aim of this pilot study is to assess the acceptability and preliminary effectiveness of the iCBT program on financial distress and associated mental health symptoms when used to treat patients with primary presentations of money worries within IAPT. A secondary aim is to explore the level of satisfaction of users with the different modules of the iCBT program.

## Materials and Methods

### Study Design

This is an open pilot study with a pre-post design that aims to examine the preliminary clinical impact of an internet-delivered program for financial difficulties and mental health as part of a routine care service delivery.

### Setting

This naturalistic study took place within the Improving Access to Psychological Therapies (IAPT) in the National Health Sservice (NHS) of England. More specifically, the study was carried out at Berkshire Healthcare NHS Foundation Trust through “Talking Therapies” Service, which is an NHS IAPT provider. IAPT is a stepped-care model for the treatment of Common Mental Health Disorders, such as depression and anxiety. Within IAPT, guided internet-delivered interventions are offered at step 2 for individuals with mild to moderate presentations of depression and anxiety.

Internet-delivered interventions in IAPT are offered with support from Psychological Wellbeing Practitioners (PWPs) who are clinicians trained in the provision of low-intensity mental health support in IAPT. Over the course of the interventions, the PWPs provide asynchronous reviews based on patients' progress on the platform, giving feedback and recommending content. Reviews are offered every 7 to 10 days and typically take an average of 15 minutes. As part of IAPT Standard Operating Procedures, PWPs provide 6 reviews on average during the course of treatment (which typically takes 8 weeks), but treatment length and number of reviews may vary depending on patient needs. This study collected data from November 2017 to May 2020. All users provided written or oral consent for their anonymized data to be used in routine evaluations for service monitoring and improvement; therefore approval from a Research Ethics Committee was not required.

### Sample

To be suitable for an internet intervention at step 2, patients are assessed by PWPs based on their willingness to use internet-delivered interventions, no suicidal or self-harm risk and having internet access. Upon initial screening, individuals who self-reported money worries as their primary presentation were assigned to the “Space from Money Worries” programme.

### “Space From Money Worries”: Intervention Development and Content

The development of *Space from Money Worries* was informed by research on the psychological links between financial difficulties and poor mental health as previously discussed. This includes the role of hopelessness and shame (28), self-esteem, active coping, and sense of agency (30). Research with Bipolar disorder has also shown the role of psychological factors, such as cognitions around achievement, dependency, and poorer mindfulness increasing compulsive spending over item (41). As well as our knowledge regarding the impact of financial difficulties on mental health, given the research suggesting a possible vicious cycle (6, 7, 9, 10), the intervention also aimed to work on factors which might lead to poor mental health impacting finances such as addressing poor financial coping and avoidance around finances which have been reported by individuals with Bipolar Disorder (10). Space from Money Worries thus aimed to tackle financial difficulties and poor mental health in an integrative fashion.

Space from Money Worries was developed to be able to help a broad spectrum and range of mental health difficulties where financial difficulties are felt to be impacting mental health and/or vice versa, ranging from those with low to moderate levels of stress, depression and anxiety to those with a diagnosis of Bipolar Disorder. It was also designed to tackle a broad range of financial issues from struggling to stick to a budget, coping with debt, managing impulsive spending, and coping with financial crises such as bankruptcy, house repossession or separation. Composite case study examples were included throughout the modules to illustrate the issues and techniques discussed.

Silvercloud health is a commerical company which has developed a number of online Cognitive Behavioral Therapy (CBT) based software packages for use in primary care. “*Space from Money Worries*” was adapted from several of SilverCloud Health's online interventions; “*Space from Stress*,” “*Space from Depression”* and “*Space from Anxiety”* which have been shown, by various randomized controlled trials, to be effective (39, 42, 43).

**Figure 1:**
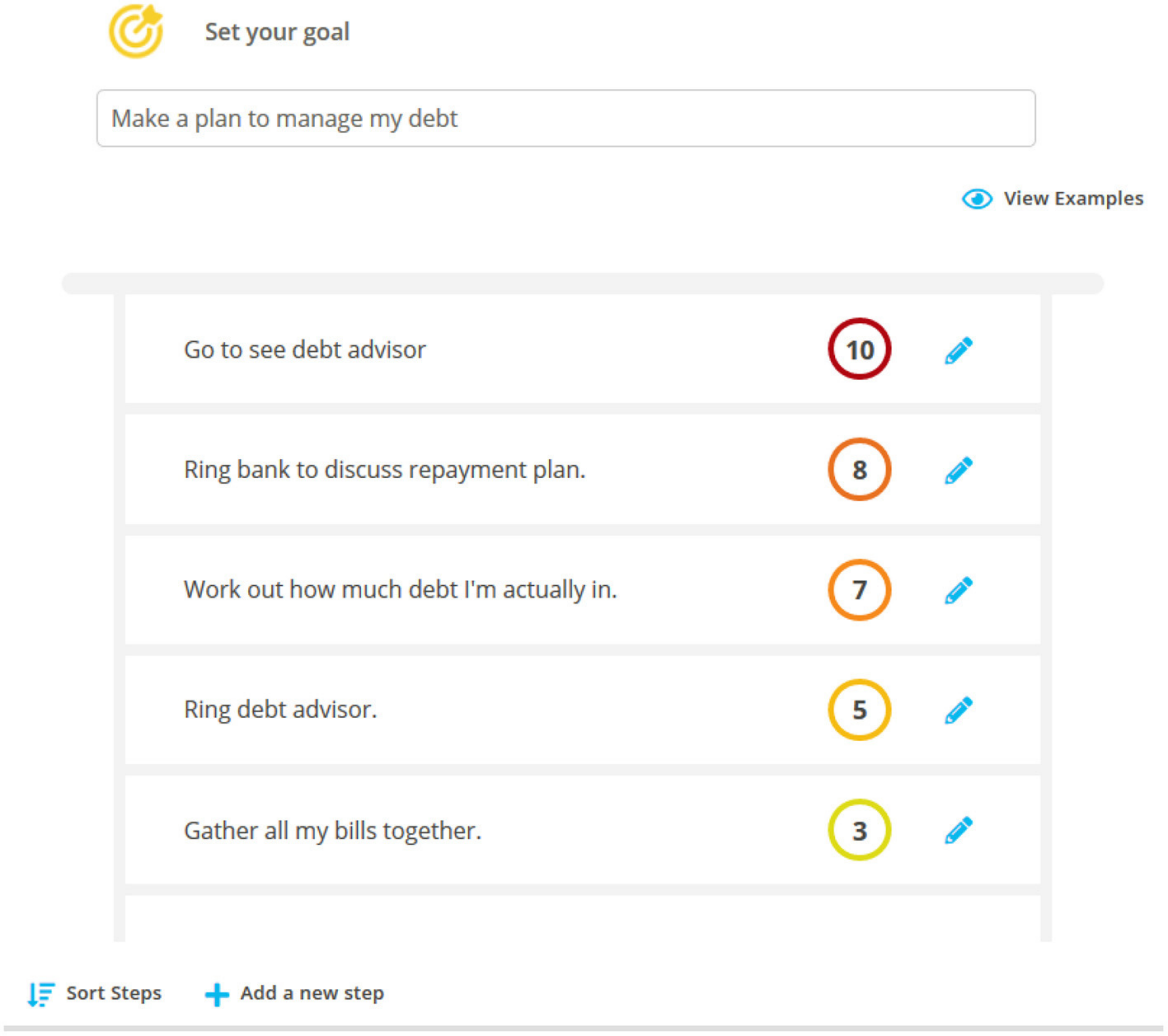
A screenshot from space from money worries: the ‘facing my financial fears' hierarchy.

**Figure 2:**
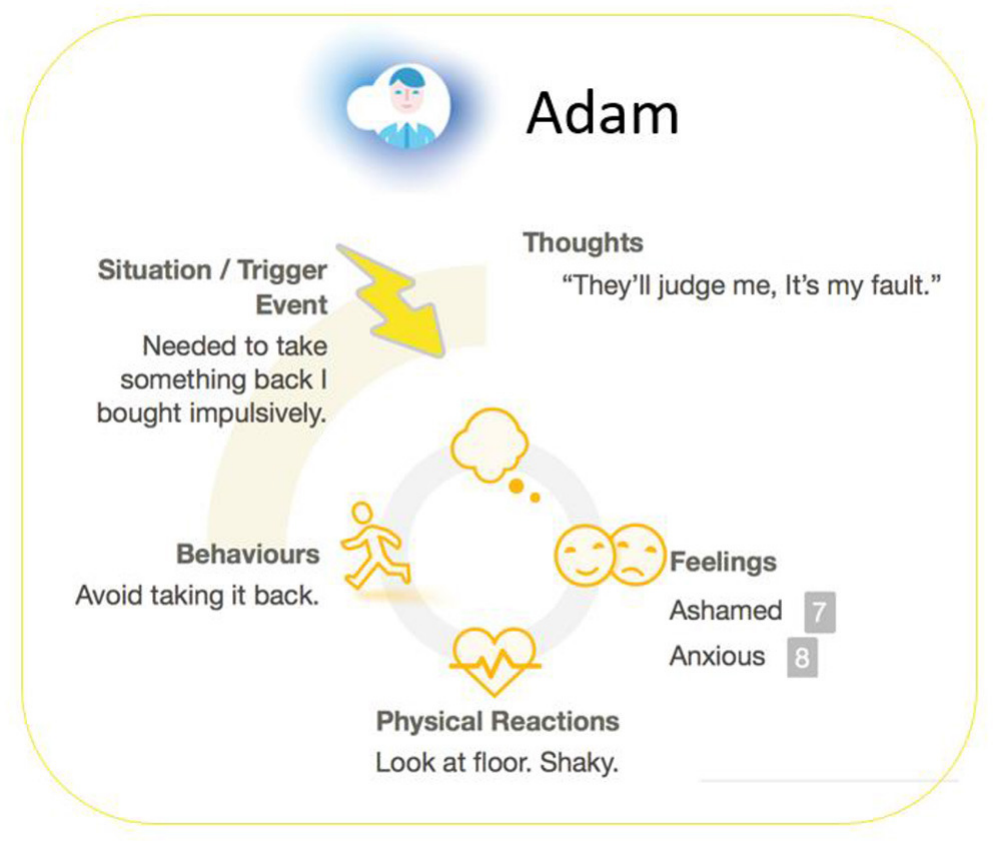
A screenshot from space from money worries: a case illustration of thought-feelings-behavior cycle around financial difficulties.

**Figure 3:**
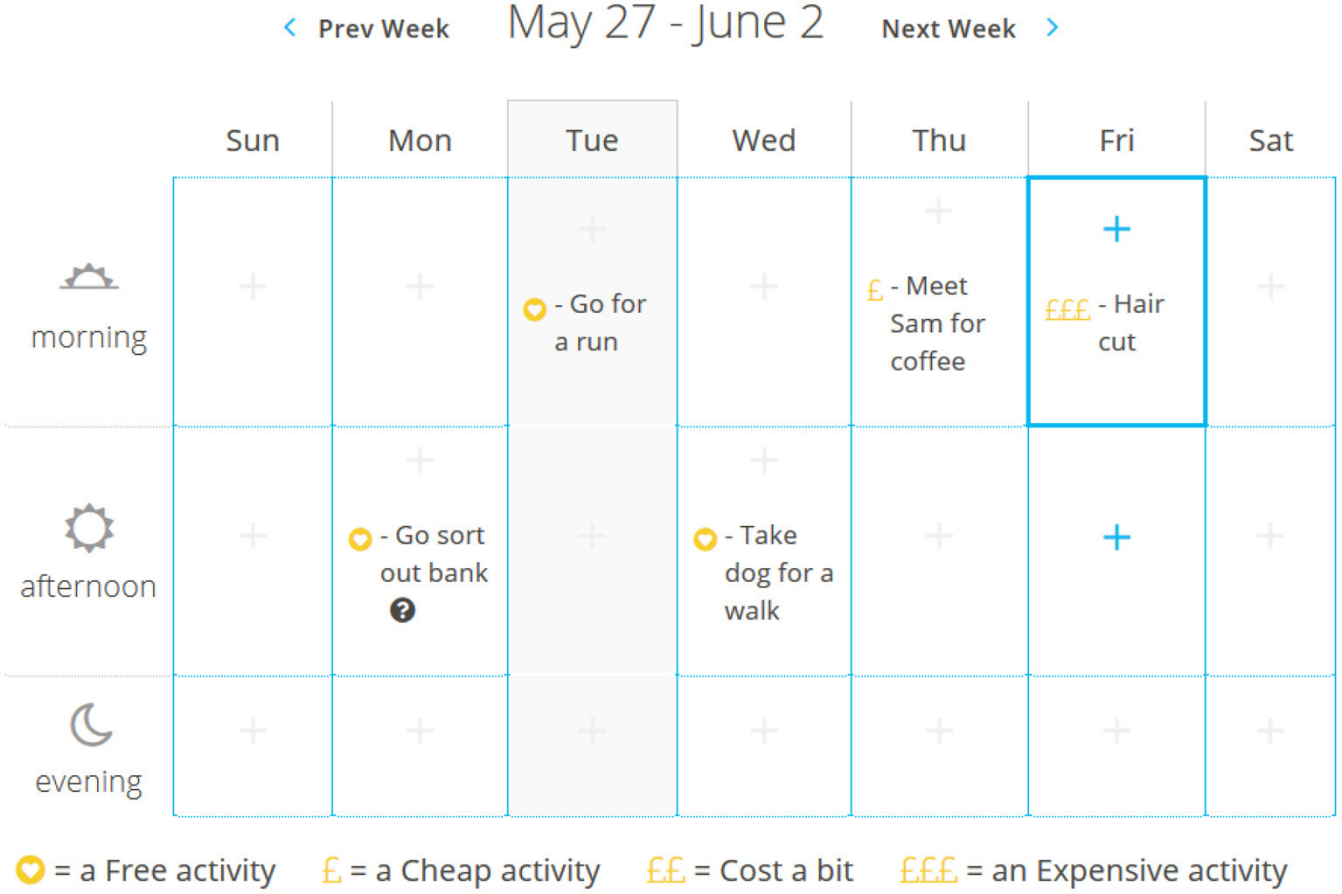
A screenshot from space from money worries: activity scheduling tool with prices included to allow for sticking to a budget.

The main therapeutic approach for psychological therapies was CBT. Table 1 outlines the 7 modules. Patients are recommended to complete one module per week, but all modules are accessible from sign up allowing room for patients to look at the content at different paces. Table 2 describes the specific therapeutic content of the intervention. Figures 1–3 demonstrate some of the content of the intervention.

**Table 1:** Overview of modules.

1. Money and mental health
2. Your thoughts about money
3. Changing your thoughts about money
4. Getting active on a budget
5. Facing your financial fears
6. Managing worry about money
7. Acceptance and hope about money difficulties
8. Getting control over impulsive spending
9. Staying financially healthy


**Table 2:** Description of specific therapy techniques used in space from money worries.

• Psychoeducation about the impact of financial difficulties on mental health and vice versa
• Thoughts-Feelings-Behavior cycle: Identifying the relationship between thoughts, emotions, and behaviors in relation to finances and vicious cycles
• Identifying cognitive distortions around finances
• Challenging cognitive distortions around finances, for example evidence to support their beliefs, and a “Cash Catastrophizing Calculator” to help work out how likely it is that their worst-case scenarios are actually going to happen
• Mood diaries with options around financial difficulties and impulsive spending to link money problems with poor mental health
• Behavioral activation and activity scheduling adapted to focus on activities which involve little or no money
• Graded exposure: Developing a “Hierarchy of Financial Fears” to help face feared and avoided financial situations and develop more proactive coping strategies
• Worry work: Strategies to reduce worry adapted around finances such as the establishment of “Money Worry Time” periods
• Mindfulness exercises: General mindfulness exercises such as a 3-minute breathing space, as well as an exercise specifically focusing on using urge surfing to resist urges to impulsively spend, and an exercise trying to help reduce rumination and fusion with money problems
• Techniques from Acceptance and Commitment Therapy (ACT) used to increase acceptance and hope around money, as well as reduce rumination and fusion around money where it might be hard to challenge thoughts as inaccurate (for example if individuals are about to be made bankrupt). A specific recorded exercise around acceptance was developed as part of this. Focusing on values was also introduced to increase hope and self-esteem during financial difficulties, and a recorded exercise around this was included
• Assertiveness Skills: These were briefly discussed to increase confidence and reduce avoidance around financial requests, such as speaking to the bank or taking an unwanted item back to a shop for a refund
• Relaxation exercises such as progressive muscle relaxation were included to try to counteract the need for “comfort spending”
• Relapse prevention focus to summarize learning and keep skills for the future
• Practical advice around financial management techniques, for example strategies to reduce the risk of impulse spending, and signposting to financial information and advice services

### Measures

- GAD-7 (44) is a 7-item measure of Anxiety symptoms which is routinely used in IAPT services as a screening and outcome measure. The GAD-7 items are based on diagnostic criteria for Generalized Anxiety Disorder. The scale ranges from 0 to 21, where higher scores reflect greater symptom severity. Cronbach's alpha at sign-up stage was 0.87.
- PHQ-9 (45) is a 9-item measure of Depression symptoms which is routinely used in IAPT services as a screening and outcome measure. The PHQ-9 items are based on the diagnostic criteria for depression. The scales ranges from 0 to 27, where higher scores reflect greater symptom severity. Cronbach's alpha at sign-up stage was 0.88.
- The In Charge Financial Distress/Financial Wellbeing scale (46): An 8-item measure of subjective financial wellbeing or distress with questions such as “*How stressed do you feel about your personal finances in general?*” (rated on a 10-point scale from “No Stress”; to “Overwhelming Stress”) and “*How often do you worry about being able to meet normal monthly living expenses?”* (rated on a 10-point scale from “Never” to “All the time”). Scores range from 8 to 80 with higher scores representing better perceived financial wellbeing. Cronbach's alpha at sign-up stage was 0.81.
- The Money and Mental Health Scale: A 9-item questionnaire was developed by the authors for the purposes of this evaluation from findings in the literature previously discussed about psychological factors in the relationship between financial difficulties and poor mental health. Questions such as “*I feel hopeless about my financial situation*” and “*I feel my financial difficulties impact my mental health”* with each questions answered: *Not at all (0), Rarely (1), Sometimes (2), Often (3), or Most or all the time (4)*, with higher scores representing a greater impact of financial difficulties on mental health and vice versa. The full scale is provided in Table 3. Cronbach's alpha at sign-up stage was 0.81.
- Module satisfaction. At the end of each of the modules, users are asked whether they perceived the modules as relevant, interesting, helpful in some way, and supportive in progressing toward their goals. The scale is composed of 4 questions that follow a 4-point Likert satisfaction scale from strongly agree, to strongly disagree. These were optional and completed anonymously online and not just for those completing the financial measures therefore the sample sizes for the different modules differ and this sample is different from that who completed the questionnaires above.

**Table 3:** Questions from the money and mental health scale.

Please answer the following in relation to the last 2 weeks
*Not at all (0) Rarely (1) Sometimes (2) Often (3) Most or all the time (4)*
1. I feel my mental health affects how much I can manage my finances.
2. I feel my financial difficulties impact my mental health.
3. I worry a lot about my debt/money situation.
4. I avoid my finances because it makes me feel anxious,
e.g., not opening bills, not ringing bank.
5. I impulsively spent more than I can afford.
6. I feel like I have no control over my financial situation.
7. I find it hard to accept my financial situation.
8. I feel hopeless about my financial situation.
9. I criticize and blame myself for my financial situation.

### Data Analysis

For the purpose of this study, an intention to treat analysis was conducted and participants that did not have a post treatment scores for all clinical measures were counted as missing. Prevalence and patterns of missing data and missing data mechanisms were explored using *t*-tests between missing and non-missing cases. Little's Missing Completely at Random (MCAR) test (47) was conducted to understand the missing pattern in the baseline and post-treatment scores and the missing data points were deemed to be “MCAR” across baseline and post-treatment scores. To handle missing data, multiple imputation by chained equation *via* “MICE” package in R was employed, undertaking 100 iterations for the imputation process, the imputation method engaged predictive mean matching (pmm) for the missing data points in the quantitative variables.

Shapiro-Wilk's test (*p* > 0.05) and a visual inspection of their histograms, normal Q-Q plots and box-plots showed that the difference between the pre/post scores for depression (PHQ-9) (W(27) = 0.90, *p* = 0.051), anxiety (GAD-7) (W(27) = 0.94, *p* = 0.156), Financial Distress/Financial Wellbeing (W(27) = 0.97, *p* = 0.610), and Money and Mental Health (W(27) = 0.97, *p* = 0.628) were approximately normally distributed and no significant departure from normality. Paired samples *t*-tests were used to analyze changes over time on measures. Within-group Cohen's *d* effect sizes were calculated utilizing the pre and post treatment change score divided by the within-group standard deviation. Stringent cut-off according to Muller (48) was used for the interpretation of the effect size whereby *d* = 0.2, 0.5, and 0.8 indicates small, medium and large effect sizes. For the financial variables, data was collected at either 4 or 8 weeks after the initial questionnaire were completed. Some participants completed both time points whilst some completed just one. For the purposes of the analyses, the last assessment available was used for all participants.

## Results

### Completion Rates

**Figure 4:**
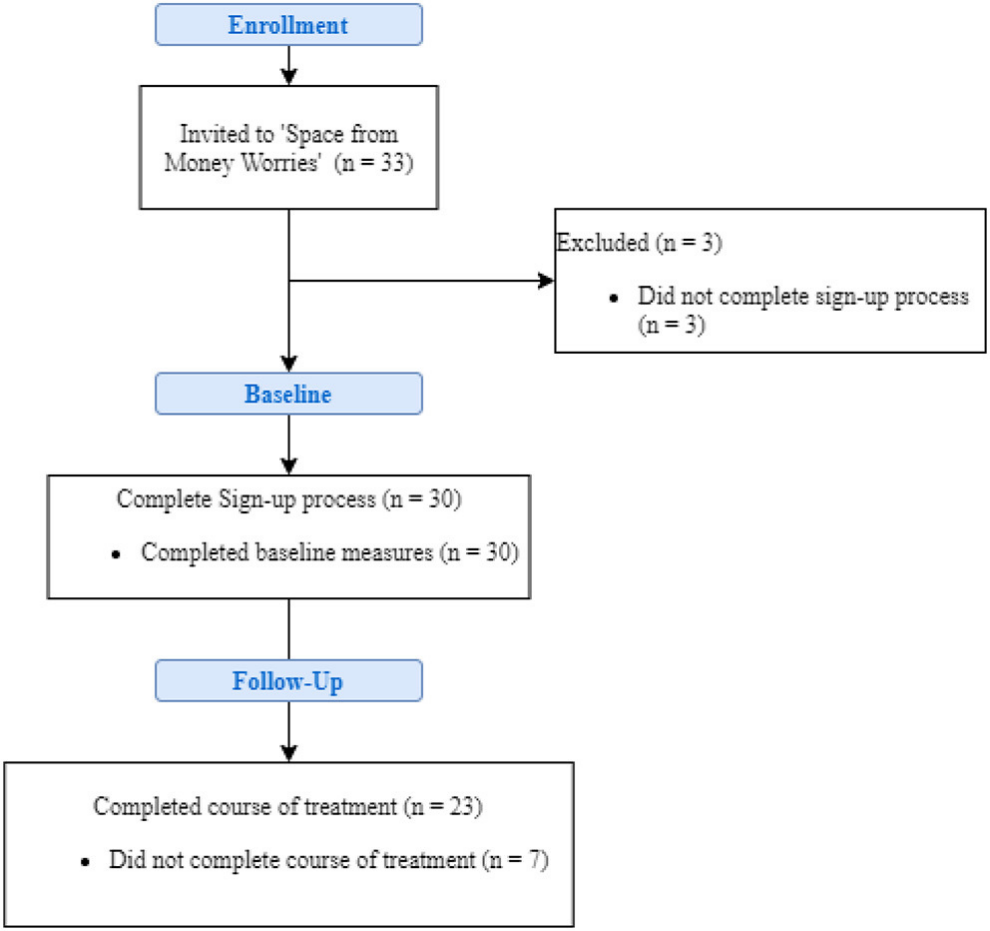
Flowchart of the study.

The thirty-three patients were invited to the program by their PWPs and from those, 30 patients successfully created an account and completed the baseline assessments. In total, 23 participants had a complete course of treatment, as per IAPT definition (i.e., minimum of two assessments). This represented an overall completion rate of 77% (*n* = 23). Figure 4 reflects the flow of participants through the trial. The average duration of treatment was 68 days.

Across the total sample, attrition rates for the completion of scales at post-treatment were at 22.2% for PHQ-9 and GAD-7. For the In Charge Financial Distress/Financial Wellbeing scale (FinDist) and the Money and Mental Health Scale (MMHS) scales, 37% of post treatment data was recorded as missing. Little's MCAR test confirmed that data was missing completely at random ($\chi_{\left(19\right)}^{2}$ = 30.8, *p* = 0.063).

### Changes in Depression and Anxiety Symptoms

A paired-samples *t*-test using intent to treat found a statistically significant reduction in PHQ-9 scores from pre (*M* = 12.22, *SD* = 5.69) to post (*M* = 6.74, *SD* = 4.04), *t*_(26)_ = 5.58, *p* < 0.001, Cohen's d (within group effect size) = 1.07 which represents are large effect size and a statistically significant reduction in GAD-7 scores from pre (*M* = 10.37, *SD* = 5.16) to post (*M* = 6.70, *SD* = 5.36), *t*_(26)_ = 3.61, *p* = 0.001, Cohen's d (within group effect size) = 0.69 which represents are medium effect size.

### Changes in Financial Variables

A paired-samples *t*-test using intent to treat found a statistically significant increase in total scores on In Charge Financial Distress/Financial Wellbeing scale from pre (*M* = 20.37, *SD* = 9.75) to post (*M* = 27.26, *SD* = 11.45), *t*_(26)_ = −3.05, *p* < 0.05. Within-group effect size was d = 0.59 which represents a medium effect size. Scores on the Money and Mental Health Scale total reduced significantly from pre (*M* = 25.37, *SD* = 5.52) to post (*M* = 22.07, *SD* = 6.84), *t*_(26)_ = 2.29, *p* < 0.05, and an effect size of d = 0.44 was observed, which represents a small effect size.

### Changes in Suicidality

Out of the 33 participants at baseline, 21.2% (*n* = 7) of these scored >0 on the PHQ-9 question 9 item “Thoughts that you would be better off dead, or of hurting yourself in some way?.” At post of these 7 participants, 42.9% (*n* = 3) scored a 0 on this.

### Feedback Data

Table 4 displays the feedback data for each module, showing that the vast majority of those completing each individual module rated it as interesting, relevant, helpful to them and supportive of them in progressing toward their goals.

**Table 4:** Feedback on the modules.

**Module**	**Number people feedback from**	**Interesting**	**Relevant to me**	**Helpful to me**	**Supporting me to make progress toward my goals**
Acceptance and hope about money	6	100%	100%	100%	100%
Changing your thoughts about money	24	100%	88%	88%	92%
Facing your financial fears	15	100%	100%	100%	93%
Getting active on a budget	20	100%	90%	95%	95%
Getting control over impulsive spending	9	100%	100%	100%	100%
Managing worry about money	9	100%	100%	78%	89%
Money & mental health (introduction)	60	78%	85%	88%	82%
Your thoughts about money	41	90%	95%	93%	95%
Staying financially healthy	9	100%	100%	100%	100%

*% Answering “Agree” or “Strongly Agree” that this module was…*.

## Discussion

This paper aimed to describe the development of *Space from Money Worries (SFMW)* and provides an initial evaluation of its acceptability and effectiveness. This program represents a unique intervention in using insights from the literature around the psychological mechanisms linking financial difficulties and poor mental health and using an online intervention to help to address these. Ratings of each module and a 77% completion rate suggest acceptability, as does the vast majority of those completing the intervention rating the program positively in terms of relevance, helpfulness, supportiveness and usefulness.

Using an initial small sample, the study also showed significantly reduced symptoms of depression and anxiety, demonstrating proof of concept that an intervention focusing solely and specifically on the relationship between poor mental health and financial difficulties may lead to improvements on broader symptoms of depression and anxiety, however due to the lack of a control group it cannot be confirmed that the changes are due to the current intervention or other variables. The finding of significantly improved perceived financial wellness supports this as a possible mechanism whereby *Space from Money Worries* may lead to improved mental health, given that the same measure has been linked significantly to greater depression and anxiety in the general population (28), as well as predicting increases in anxiety and stress over time in those with Bipolar Disorder (13). However, future research with a larger sample allowing for mediation analysis will help confirm if this is indeed a mechanism of therapeutic action for the current intervention.

The money and mental health measure was also shown to significantly improve following SFMW, suggesting that participants felt that this reduced how much financial difficulties impacted their mental wellbeing and vice versa. However again caution is required around causality due to lack of a control group. This is in line with research showing the impact of financial difficulties such as debt on mental health (4), and research on psychological factors which link financial difficulties and mental health such as stress, concern about debt, shame and hopelessness (24, 28, 49). This improvement was significant even with an intent to treat analysis though the effect size was small. Although there was a good Cronbach's alpha for the measure in this sample, it has not been formally developed and evaluated and therefore these results need to be interpreted with caution.

There was a reduction in the number of participants reporting suicidal ideation following the intervention. Research has shown increased suicidal ideation for those in financial distress (23, 29). However, due to the small proportion of individuals reporting suicidal thoughts in this sample further research is required to see if an intervention such as SFMW can reduce suicidality. Given that suicidality is often an exclusion criteria for IAPT, an evaluation of this intervention with a sample with a greater proportion of suicidality is required.

This study is limited by a small sample size and no control group. The clinical and demographic profile of the sample was also not recorded, which prevented understanding the profile of participants and if it had differential effects in specific subgroups. Feedback about the modules was from varying samples and a different sample to those who completed the questionnaires. Further research is needed using a randomized controlled trial design with a larger sample and longer follow-up to determine the effectiveness of *Space from Money Worries* when compared to other interventions which are not specifically focusing on financial difficulties, such as broader online CBT based interventions. It would also be helpful to assess whether addressing the psychological mechanisms specifically, has a greater impact than non-psychological interventions such as signposting to debt advice services: “job club” interventions have been found to improve depression in those who are unemployed (32). However, other research trying to provide debt counseling for depression in primary care has had to stop due to poor recruitment, with the authors concluding that this highlights the “*need to widen the focus of research investigation to determine the mechanisms of psychological distress in the context of debt*” (50), suggesting that a targeted psychological intervention such as the one provided here may be necessary. Given the role of avoidance of financial difficulties when individuals are depressed (10), future research could examine whether *Space from Money Worries* leads to improved engagement with formal debt advice services in those with mental health problems.

In conclusion, this preliminary study has suggested that a targeted online psychological intervention is acceptable and appears to be effective for those with financial difficulties and poor mental health which are impacting one another, though replication with a control group is required. Given the finding that those in debt are more than three times more likely to have a mental disorder compared to those not in debt (4), this could have potential at a public health level.

## Data Availability Statement

The raw data supporting the conclusions of this article will be made available by the authors, without undue reservation.

## Ethics Statement

Ethical review and approval was not required for the study on human participants in accordance with the local legislation and institutional requirements. Written informed consent for participation was not required for this study in accordance with the national legislation and the institutional requirements.

## Author Contributions

TR, DR, CE, and DH developed the initial study design. AE, AA, and TR analyzed the data. All authors contributed to writing up the manuscript for publication.

## Funding

This study was funded by SilverCloud Health and has been possible thanks to the partnership with Berkshire Healthcare NHS Foundation Trust (England). The funder was not involved in study design or data collection, but provided advise in the analysis, preparation of the manuscript and the decision to submit it for publication. TR has received consultancy payments from SilverCloud Health for the development and evaluation of the Space from Money Worries program. He also receives royalties from its use.

## Conflict of Interest

TR works for Richardson Psychological Consultation which was contracted by SilverCloud Health to work on developing and researching outcomes from Space from Money Worries. He receives royalties from Space from Money Worries use. DR, CE, AA, AE, and DH are employees of SilverCloud Health, developers of computerized psychological interventions for depression, anxiety, stress, sleep, resilience, and comorbid long-term conditions. AE and DR are also researchers with the E-Mental Health Research Group of Trinity College Dublin TCD and adhere to both the research ethics frameworks and policy on good research practice of TCD.

## Publisher's Note

All claims expressed in this article are solely those of the authors and do not necessarily represent those of their affiliated organizations, or those of the publisher, the editors and the reviewers. Any product that may be evaluated in this article, or claim that may be made by its manufacturer, is not guaranteed or endorsed by the publisher.
